# Chemical Composition and Microstructural Morphology of Spines and Tests of Three Common Sea Urchins Species of the Sublittoral Zone of the Mediterranean Sea

**DOI:** 10.3390/ani10081351

**Published:** 2020-08-04

**Authors:** Anastasios Varkoulis, Konstantinos Voulgaris, Stefanos Zaoutsos, Antonios Stratakis, Dimitrios Vafidis

**Affiliations:** 1 Department of Ichthyology and Aquatic Environment, Nea Ionia, University of Thessaly, 38445 Volos, Greece; dvafidis@uth.gr; 2 Department of Energy Systems, University of Thessaly, 41334 Larisa, Greece; szaoutsos@teilar.gr; 3 School of Mineral Resources Engineering, Crete Technical University of Crete, 73100 Chania, Greece; astratak@mred.tuc.gr

**Keywords:** sea urchins, Mediterranean Sea, ultrastructure, powder X-ray diffraction, structural morphology

## Abstract

**Simple Summary:**

*Arbacia lixula*, *Paracentrotus lividus* and *Sphaerechinus granularis* play a key role in many sublittoral biocommunities of the Mediterranean Sea. However, their skeletons seem to differ, both morphologically and in chemical composition. Thus, the skeletal elements display different properties, which are affected not only by the environment, but also by the vital effect of each species. We studied the microstructural morphology and crystalline phase of the test and spines, while also examining the effect of time on their elemental composition. Results showed morphologic differences among the three species both in spines and tests. They also seem to respond differently to possible time-related changes. The mineralogical composition of *P. lividus* appears to be quite different compare to the other two species. The results of the present study may contribute to a better understanding of the skeletal properties of these species, this being especially useful in predicting the effects of ocean acidification. More specifically, since the skeleton plays a key role for the survival of sea urchins in general, a potential change in any skeletal structure, either morphologically or chemically, may affect these organisms directly while also affecting their ecosystem indirectly.

**Abstract:**

In the Mediterranean Sea, the species *Arbacia lixula*, *Paracentrotus lividus* and *Sphaerechinus granularis* often coexist, occupying different subareas of the same habitat. The mechanical and chemical properties of their calcitic skeletons are affected both by their microstructural morphology and chemical composition. The present study describes the main morphologic features and the possible temporal differences in elemental composition of the test and spines of the three species, while also determining the molar ratio of each element of their crystalline phase. Scanning electron microscopy showed major differences in the ultrastructure of the spines, while minor differences in the test were also noticed. More specifically, the spines of all three sea urchins possess wedges, however *A. lixula* exhibits bridges connecting each wedge, while barbs are observed in the wedges of *S. granularis.* The spines of *P. lividus* are devoid of both microstructures. Secondary tubercles are absent in the test of *A. lixula*, while the tests and spines of all three species are characterized by different superficial stereom. Energy dispersive x-ray spectroscopy detected that Ca, Mg, S, Na and Cl were present in all specimen. Mg and Mg/Ca showed significant differences between species both in test and spines with *S. granularis* having the highest concentration. The spines of *P. lividus* exhibited lowest values between all species. Differences between spines and test were observed in all elements for *P. lividus* except S. *A. lixula* exhibited different concentrations between test and spines for Ca, Mg and Mg/Ca, whereas *S. granularis* for Mg, Cl and Mg/Ca. Finally, temporal differences for Ca were observed in the test of *P. lividus* and the spines of *S. granularis*, for Mg in test of *S. granularis*, for S in the spines of *A. lixula* and the test and spine of *S. granularis*, for Na in the test of *P. lividus* and *A. lixula* and for Cl and Mg/Ca in the test *P. lividus.* Powder X-ray diffractometry determined that, out of all three species, the spines of *P. lividus* contained the least Mg, while the test of the same species exhibited higher Mg concentration compared to *A. lixula* and *S. granularis*. The current study, although not labeling the specimens attempts to estimate potential time-related elemental differences among other results. These may occur due to changes in abiotic factors, probably water temperature, salinity and/or pH. Divergence in food preference and food availability may also play a key role in possible temporal differences the skeletons of these species

## 1. Introduction

Biomineralization in marine invertebrates is a rather common phenomenon, where organisms produce minerals in order to enhance, strengthen and support existing tissue (i.e., teeth, shells, spines). These biominerals tend to have different properties in comparison to their abiotic counterparts [1]. The skeleton of echinoderms is primarily made up of Mg-rich calcite [(Mg, Ca)(CO_3_)], with small amounts of stable amorphous calcium carbonate (ACC), water and intra-crystalline organic molecules [2]. In contrast to other invertebrates, echinoderms possess a skeleton, which generally contains interconnecting cavities and much open space [3]. This is most noticeable when observing the test and spines of sea urchins.

Sea urchins are also considered drivers of the ecosystem and are important grazers in many sublittoral communities [4,5,6,7,8]. They can remove seagrass meadows and kelp forests, turning them into coralline barrens and thus play an important role in shaping the structure and functioning of many benthic marine biomes, especially rocky-reef ecosystems [9,10]. In the Mediterranean Sea, *Paracentrotus lividus* (Lamark, 1816), *Arbacia lixula* (Linnaeus, 1758) and *Sphaerechinus granularis* (Lamark, 1816) are some of the most common sea urchin species, often coexisting in the same habitat. It has been shown that the grazing activity of these three species can actually alternate the type of an ecosystem both separately and combined [11,12,13,14]. However, not only do they utilize different defensive adaptations, they also have different ecological and biologic features.

The lightweight property of the test originates from the fenestrated structure and relatively high porosity [15]. Thus, it would be thought that the dome shaped structure combined with the single- crystal calcite would make the test rather weak. However, it is far stronger than expected, because of the organic nature of biominerals. It is in fact many times stronger than calcareous rocks and mollusk shells [13]. Normal calcite is extremely brittle, in contrast to the strength to weight ratio of biomineralized calcite in sea urchins, which is by far higher compared to any other type of calcite [16,17].

The main weakness of a high-magnesium (Mg) calcite endoskeleton is that it may be vulnerable to CO_2_-driven ocean acidification, however there are studies that support that the epidermis and structural properties of the crystal can mitigate these effects [18,19,20]. The solubility of Mg-calcite increases with increase of Mg content in the crystal [21,22,23]. Calcite that contains more than approximately 12% weight fraction MgCO_3_ is considered more soluble than aragonite [20]. Since the sea urchin skeleton is rich in Mg^2+^ content, which in turn is responsible for the high solubility of the mineral form [20,23,24], sea urchins are likely to be particularly vulnerable to the effects of ocean acidification. The fact that the skeletal elements (test, teeth, etc.) play a key role [24,25,26,27], in the survival of sea urchins could make this taxon even more susceptible to the effects of ocean acidification, since it may increase the pressure of predation on these organisms.

It is reported that depending on the environment and other factors such as the vital effect, different species have different Mg concentrations. For example, *Lytechinus variegatus* and *Heterocentrotus mammilatus* being warm water species, have a higher amount of Mg substituted for Ca into their skeletal parts in the range of 4 mol% to 10 mol%, while cold water species, like the extreme example *Sterechinus antarcticus*, have only about 2 mol% to 3 mol% Mg content [28]. However, the Mg content of different skeletal elements of the same species also varies. For example, the Mg concentration of the test of *L. variegatus* ranges from 9.6 wt% to 11.3 wt% MgCO_3_, while the spines of the same species only contain 5.5 wt% to 8.3 wt% MgCO_3_. Finally, the mouth parts of the same species exhibit Mg concentrations of 8.3 wt%–13.4 wt% MgCO_3_ [16,23,29,30,31,32].

All three species utilize their test and spines as their main defensive skeletal structures, but their shape and chemical composition differ. Thus, their tolerance against the effects of ocean acidification may vary. The aim of the present study is to determine test and spines chemical composition of these three common Mediterranean species. This will help to indirectly evaluate the extent of vulnerability to ocean acidification for the skeleton of each species, but, more important, to assess the danger, that climate change poses, regarding the sublittoral benthic communities of the Mediterranean.

## 2. Materials and Methods

### 2.1. Sampling Site and Study Period

**Figure 1:**
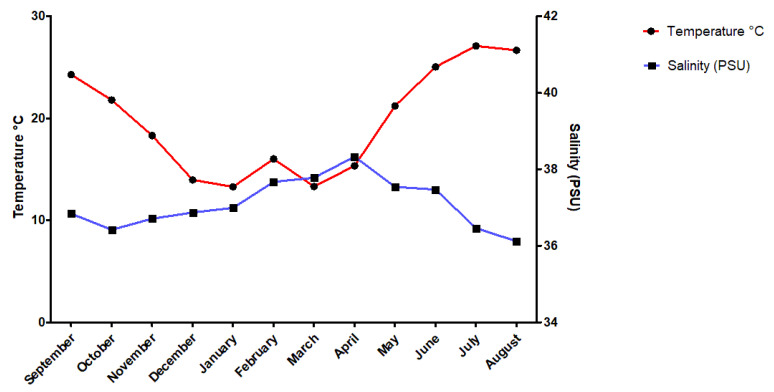
Diagrams showing monthly variations of temperature (°C) and salinity (PSU) in the sampling site.

The study was performed on three species of sea urchins, namely, *Paracentrotus lividus* (Lamarck, 1816), *Sphaerechinus granularis* (Lamarck, 1816) and *Arbacia lixula* (Linnaeus, 1758) and took place in the Gulf of Pagasitikos, which is located in the central-western region of the Aegean Sea. It is a semi -enclosed shallow water basin (mean depth is 69 m) in which the water masses are cold and homogeneous in winter (12.6 °C) and highly stratified in the summer (27.5 °C) [32]. In the sampling area (Agios Stephanos 39°18’12.7” N 22°56’26.7” E) the seabed consists mainly of soft substrate with sparse meadows of *Zostera marina* (Linnaeus, 1753) and patches of rocks, dominated by communities of photophilic algae. Samples were collected seasonally (November 2017, February 2018, May 2018 and August 2018) by scuba diving at depths between 1 and 12 m. Concurrently, the seawater temperature and salinity was recorded monthly with an autographic conductivity temperature depth recorder, CTD (SeaBird, Bellevue, WA, USA), (Figure 1).

### 2.2. Microstructural Morphology and Elemental Analysis

**Figure 2:**
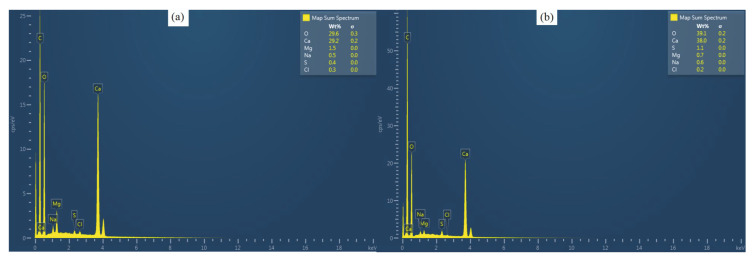
EDS spectra of A. lixula (**a**) test and (**b**) spine.

Five individuals of each species from each sampling date were examined they were first dissected in order to acquire spines from the middle section of the interambulacral zones and interambulacral plates near the peristome. The plates and spines were then cleaned of internal organs using a 1% bleach treatment in distilled water for 30 min and dried at 60 °C for 3 days [33]. Specimens were coated in carbon by a Q150R Plus-rotary pumped coater carbon thread evaporator, reducing picture clarity in comparison to a gold coating, but enabling Energy-Dispersive X-Ray Spectroskopy (Figure 2). The samples were examined and imaged by SEM JEOL JSM 6510. For the cross-section of the spines, the middle part of the shaft was selected. Five observations for each species were carried out to determine the number of wedges. To study the chemical composition of the spines and tests, an EDS analysis was carried out by a JEOL JSM 6510 scanning electron microscope, equipped with an Oxford Link ISIS 300 system. Each measurement lasted 240 sec and was made in a 0.10^2^ mm area. The characterization of the various stereom was carried out according to Smith, 1980.

### 2.3. X-ray Powder Diffraction Analysis

An X-ray diffraction analysis (XRD) of the studied samples was performed on a D8 Advance—BrukerAXS diffractometer using CuKα radiation in order to determine the crystalline phase both quantitatively and qualitatively. Interambulacral plates and spines from all individuals of each species were triturated in an agate mortar until a fine state (<40 μm). Afterwards 1 g of powder was placed in a standard cuvette and was mounted in the X-ray diffractometer. Measurements were carried out by a LynxEye detector with Ni-filter, operated at the voltage of 35 kV and the intensity of 35 mA, at a 2θ scanning range of 4–70°; analyses were made at a step of 0.02° and a speed of 0.2 s per step. The evaluation of data were carried out with the Diffracplus EVA–BrukerAXS software. Identification of the experimental data were performed by fitting the diffraction pattern using the JCPDS (joint committee on powder diffraction standard) database.

### 2.4. Statistical Analysis

Data of the elemental percentage compositions of each species were checked for normality (Kolmogorov–Smirnov) analysis of variance (one-way ANOVA) was used both for temporal elemental comparisons and for elemental comparison between species, followed by suitable post hoc tests (Tukey), where needed [34]. Data that did not follow a normal distribution or were not homogenous were analyzed by the Kruskal–Wallis one-way-analysis. Significant differences were further examined using post hoc tests (Dunn’s test). The variance of the group means for ANOVA (F value) and the variance of the group means for Kruskal–Wallis (H value) are reported together with the probability value (*p*), in order to give a more detailed observation regarding the variation among group means. For the elemental comparisons between tests and spines of each species Mann–Whitney U test was used. Statistical analyses were performed using the GraphPad Prism statistics software package (GraphPad Prism 5.0, GraphPad Software, San Diego, CA, USA).

## 3. Results

### 3.1. Morphology of Tests and Spines

#### 3.1.1. Arbacia Lixula

**Figure 3:**
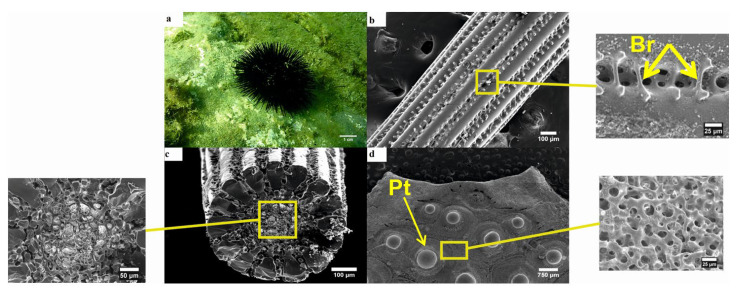
Black sea urchin *Arbacia lixula*. (**a**) General in situ view of the aboral side; (**b**) longitudinal section of the shaft of the spine (×100), with enlarged image showing bridges connecting two wedges (×650); (**c**) transverse section of the spine (×160), with enlarged image showing the porous zone (×350); (**d**) section of the test from the peristomal region (×16), with enlarged image showing the stereom of the outer surface of the plate (×600). Br—bridges, Pt—primary tubercles.

The black sea urchin is usually attached on rocks or other hard substrate and does not use any type of cover (Figure 3a). Externally the spines are made of radial and longitudinal dense wedges, also called septa, which encircle the inner central stereom. The wedges are connected with each other via another type of skeletal microstructure called bridges (Figure 3b). The cross-section of the spine is a single layer of wedges along the inner surface, while the rest of the area is covered by the porous zone. The inner stereom of the spines of *A. lixula* seems to be of labyrinthic type, with irregular meshwork, while the outer cortex consists of solid imperforate stereom without pores (Figure 3c). The plates of the test bear the primary tubercles, where the base of the primary spines fit, like a ball—socket joint. Secondary tubercles seem to be absent in this species. As Smith (1980) [15] noted the outer surface of the plate is covered by a well-developed, compact, perforate stereom layer. Most of the pores, which are cylindrical shaped, seem to be located perpendicularly to the surface, thus the superficial stereom layer is characterized as mainly simple perforate (Figure 3d). The perforate stereom layer generally is a fairly thick layer, usually thicker than the average maximum diameter of the pores that perforate it and the main characteristic is that the pores are unbranched and mainly perpendicular to the surface, as in the case of the outer stereom surface of *A. lixula*.

#### 3.1.2. Paracentrotus Lividus

**Figure 4:**
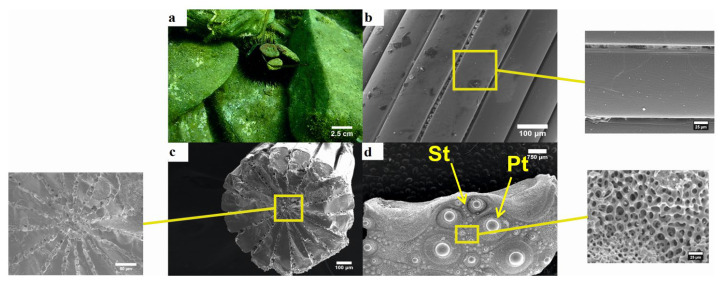
Purple sea urchin *Paracentrotus lividus*. (**a**) General in situ view of the aboral side; (**b**) longitudinal section of the shaft of the spine (×100), with enlarged image showing a wedge of the spine (×550); (**c**) transverse section of the spine (×100), with enlarged image showing the inner part of the spine. Notice that no porous zone is present; (**d**) section of the test from the peristomal region (×16), with enlarged image showing the stereom of the outer surface (×600). St—secondary tubercles; Pt—primary tubercles.

The purple sea urchin is typically found on hard substrate (e.g., rocks), where it covers itself with various materials (e.g., pebbles, mollusk shells, etc.) by holding them between its spines (Figure 4a). The wedges of the spine seem to be very close to each other, with very little to no gap in between. Furthermore, no skeletal microstructures are observed on the wedges, which are in turn smooth in texture (Figure 4b). On the transverse view, the 17 triangular wedges seem to take up all the inner space of the spine, while the porous zone seems to be relatively limited (Figure 4c). Multiple tubercles are visible on the plates of the test, where the spines are attached with a ball–socket type connection, while secondary tubercles seem to cover much of the free space of the test. The outer surface of the test is covered by a thin layer of unorganized mesh of trabeculae, which forms a dense labyrinthic stereom. It appears as a three-dimensional tangle of interconnected trabeculae. These trabeculae are rather thick, while all intersections have prominent thorns. (Figure 4d). The labyrinthic stereom is the main architectural design used by most echinoids. The primary characteristic is the isotropic arrangement of the trabeculae, which ensures that the stereom is equally strong in all directions.

#### 3.1.3. Sphaerechinus Granularis

**Figure 5:**
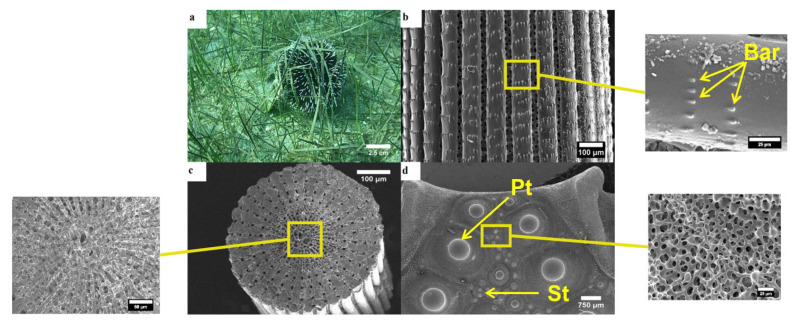
The purple sea urchin *Sphaerechinus granularis*. (**a**) General in situ view of the aboral side; (**b**) longitudinal section of the shaft of the spine (×100), with enlarged image showing rows of barbs on a wedge (×1100); (**c**) transverse section of the spine (×140), with enlarged image showing the porous zone (×400); (**d**) section of the test from the peristomal region (×16), with enlarged image showing the stereom of the outer surface. Bar—barbs, Pt—primary tubercles, St—secondary tubercles.

The violet sea urchin is typically found on soft substrate (e.g., sand), many times concealed in algal meadows, camouflaging itself with soft materials like leaves or algae (Figure 5a). The inner stereom of the spines is surrounded by wedges, which are characterized by rough surface, where rows of 3–5 barbs can be distinguished (Figure 5b). The transverse view unveils the porous zone, which is encircled inside the rectangular wedges. The outer stereom seems to process parallel galleries running in one direction and no pore alignment is parallel to this direction. These galleries can be characterized as parallel trabeculae rods interconnected with struts. The inner stereom consists of “tube-like” structures with seemingly equally arranged pores. The surface is perforated by numerous closely spaced pores with a relatively large diameter. Thus, the superficial layer of the stereom seems to be composed of the radiating layer is galleried, whereas the medulla is laminar. There are 32 wedges around the porous zone, which extend over half of the inner surface of the spine, forming four concentric layers (Figure 5c). The plates of the test contain tubercles where the spines are attached, while secondary tubercles are also visible. The outer layer of the plate consists of a mesh of trabeculae, which creates a perforate stereom layer between tubercles. This layer seems to be thicker than the diameter of the pores, which perforate it. The pores are circular shaped and irregularly arranged. Since they are sinuous and branched an irregular perforate stereom occurs (Figure 5d).

### 3.2. Elemental Analysis

#### 3.2.1. Calcium

Statistical differences between test and spines were observed in *A. lixula* (*p* = 0.03) and *P. lividus* (*p* = 0.0003), with the spines containing higher concentrations of calcium. Ca concentrations between test and spine showed no differences for *S. granularis*.

##### Spines

**Figure 6:**
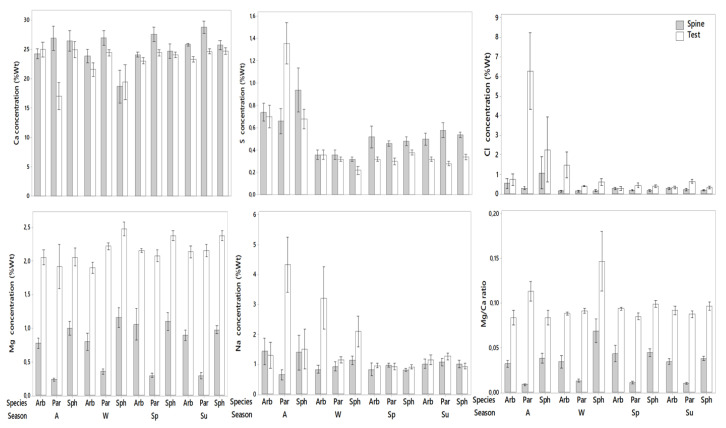
Temporal wt% concentrations of Ca, Mg, S, Na, Cl and Mg/Ca ratio in the spines and tests of the three species.

Comparison between species showed statistical differences between *P. lividus* and the other two species (Kruskal–Wallis H test, H = 13.767, *p* = 0.001) (Table 1). Temporal differences in the Ca content of the spines were only observed in *S. granularis* (F = 3.553, *p* = 0.038). Highest values occurred in samples collected in summer (45.88 wt% ± 2.28 wt%), while lowest in autumn (34.23 wt% ± 9.5 wt%) (Table 2; Figure 6). The spines of both *A. lixula* and *P. lividus* showed no significant temporal differences.

##### Test

No statistical differences were observed in the Ca content between the three species (Table 1). The test of *P. lividus*, exhibited significantly lower values in Autumn (F = 9.036, *p* = 0.001), compared to all others with a value of 21.09 wt% ± 16.89 wt% (Table 2; Figure 6). No temporal differences were observed in Ca of the test for *A. lixula* and *S. granularis*.

#### 3.2.2. Magnesium

The tests of all species showed significantly more Mg content compared to the spines (*p* = 0.0001 for all species).

##### Spines

The Mg content of spines was significantly different among the three species (Kruskal–Wallis H test, H = 39.674, *p* = 0.0001). *P. lividus* exhibited significantly lower values compared to the other two species with a value of 0.31 wt% ± 0.09 wt% (Table 1). No statistical differences were observed in the temporal concentrations of the three species (Table 2; Figure 6).

##### Test

The three species exhibited significant differences in Mg content (Kruskal–Wallis H test, H = 8.19, *p* = 0.0168). Highest values were observed in the test of *S. granularis* (2.44 wt% ± 0.6 wt%) and lowest in *P. lividus* (2.15 wt% ± 0.66 wt%). Temporal differences occurred only for *S. granularis* (F = 6.206, *p* = 0.0053) (Table 2). More specifically, samples collected in autumn exhibited lower values (1.72 wt% ± 0.73 wt%) compared to the others (Figure 6). *A. lixula* and *P. lividus* showed no temporal differences regarding the Mg content in the test.

#### 3.2.3. Sulfur

The spines seem to have higher S concentrations compared to the tests in all three species (*A. lixula*, *p* = 0.025; *P. lividus*, *p* = 0.017; *S. granularis*, *p* = 0.0001).

##### Spines

No significant differences were observed among the three species (Table 1), while temporal differences occurred in *A. lixula* (F = 4.847, *p* = 0.013) and *S. granularis* (F = 11.311, *p* = 0.01) (Table 2). Lowest values for *A. lixula* were observed in samples collected during winter (0.51 wt% ± 0.11 wt%), while highest occurred in autumn (1.002 wt% ± 0.17 wt%). *S. granularis* showed lowest S concentrations during samples collected in winter (0.49 wt% ± 0.05 wt%) and highest during summer (0.76 wt% ± 0.06 wt%) (Figure 6). *P. lividus* exhibited no significant temporal differences.

##### Test

The three species showed no statistical differences regarding the S concentration in their tests. Temporal differences were observed only in *S. granularis* (Kruskal–Wallis H test, H = 8.056, *p* = 0.044) with lowest values occurring during the sample collection winter (0.32 wt% ± 0.1 wt%) and highest during spring (0.54 wt% ± 0.05 wt%) (Table 2; Figure 6). No significant temporal differences were observed in the S content of the test of *A. lixula* and *P. lividus*.

#### 3.2.4. Sodium

Statistical differences between test and spines were only observed in *P. lividus* (*p* = 0.002) with the test containing higher Na concentration than the spine. No significant differences were observed among the three species regarding both the tests and spines (*p* = 0.57).

##### Spines

There were no significant temporal differences in the Na concentration of the spines of all three species (Table 2; Figure 6).

##### Test

Temporal differences occurred both in *A. lixula* (F = 3.546, *p* = 0.03) and *P. lividus* (F = 11.368, *p* = 0.0003) (Table 2). *A. lixula* exhibited higher values (3.45 wt% ± 2.22 wt%) in the samples that were collected in winter compared to the others. Post hoc test showed that highest values for *P. lividus* occurred during autumn sampling (2.79 wt% ± 1 wt%) (Figure 6), while no significant differences were observed among the other sampling periods. *S. granularis* showed no significant temporal differences in the Na concentration of the test.

#### 3.2.5. Chlorine

*P. lividus* (*p* = 0.0001) and *S. granularis* (*p* = 0.0002) showed statistical differences between test and spines, with the test exhibiting higher concentrations compared to the spines in both species, while Cl content of *A. lixula* did not vary between test and spines. No significant differences were observed between species in the spines (*p* = 0.13) or the test (*p* = 0.09) (Table 1).

##### Spines

No significant differences were observed in the three species regarding the temporal effects. (A. lixula: F = 1.503, *p* = 0.25; *P. lividus*: Kruskal–Wallis H test, H = 3.387, *p* = 0.33; S. granularis: Kruskal–Wallis H test, H = 0.42, *p* = 0.93) (Table 2; Figure 6).

##### Test

*P. lividus* was the only species that showed significant temporal differences (F = 16.404, *p* = 0.0001) (Table 2). Higher values were recorded in samples collected during autumn (3.06 wt% ± 1.11 wt%) compared to all others (Figure 6). *A. lixula* and *S. granularis* exhibited no temporal differences regarding the Cl concentration of the test.

#### 3.2.6. Mg/Ca Ratio

The Mg/Ca ratio was significantly higher in the tests of all three species compared to the spines (*A. lixula*, *p* = 0.0001; *P. lividus*, *p* = 0.0001; *S. granularis*, *p* = 0.0001).

##### Spines

Significant differences were observed among the three species (Kruskal–Wallis H test, H = 40.538, *p* = 0.0001) (Table 1). *P. lividus* exhibited lowest values compared to the other two species, with a value of (0.006 wt% ± 0.002 wt%). No temporal differences were observed for all species (Table 2; Figure 6).

##### Test

The Mg/Ca ratio varied significantly among the three species (Kruskal–Wallis H test, H = 6.08, *p* = 0.047) (Table 1). More specifically, statistical differences were observed between *A. lixula* (0.054 wt% ± 0.005 wt%) and *S. granularis* (0.066 wt% ± 0.025 wt%), but not between *P. lividus* and the other two species. Temporal differences occurred only in *P. lividus* (F = 12.576, *p* = 0.0002) (Table 2). Highest values were observed during the samples that were collected in autumn (0.08 wt% ± 0.01 wt%), compared to the other three s (Figure 6). No significant temporal differences were observed in the Mg/Ca ratio of the test of *A. lixula* and *S. granularis*.

### 3.3. X-ray Powder Diffraction

**Figure 7:**
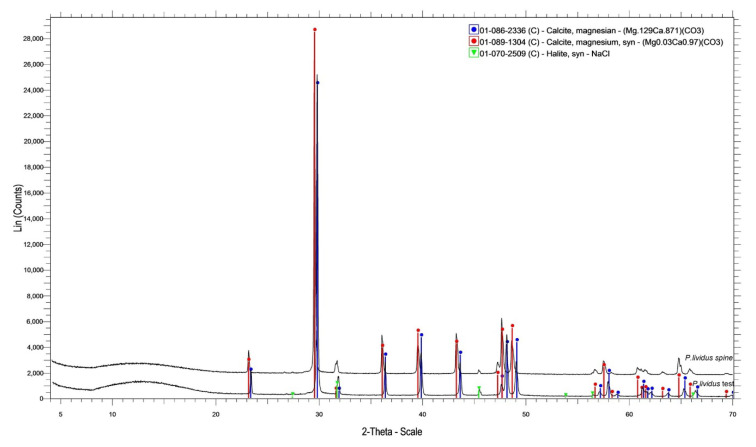
Representative powder XRD pattern of test and spines of *P. lividus.*

The phase composition of the samples is given in Table 3. It was shown that all the spines and tests of all three species consist of some form of magnesian calcite and a small percentage of halite (NaCl) (Figure 7, Table 3). *A. lixula* and *S. granularis* had roughly the same quantities of magnesium in their crystalline phases both in the spine and the test. *P. lividus* had the highest values of magnesium in the test and the lowest in the spine, more specifically double the amount of magnesium was found in the test of *P. lividus*, whereas half of it was found in the spines, compared to *A. lixula* and *S. granularis*. Halite took up 3% of the composition of all samples except for the spines of *S. granularis*, which consisted of 4% NaCl.

## 4. Discussion

The three investigated species are commonly found in the Mediterranean Sea, often coexisting in the same habitat [35]. However, *S. granularis* was mainly found in soft substrate in contrast to *A. lixula* and *P. lividus*, which were attached on hard substrates (mainly rocks) (Figure 3a, Figure 4a, Figure 5a). Various morphologic differences were observed regarding their ultrastructure, both in their spines and tests. The spines of *P. lividus* seem to be deprived of any skeletal microstructures (e.g., bridges, barbs) and are characterized by a smooth texture, whereas both other sea urchins are acquired with spines, which are characterized by rough textures due to bridges between wedges (*A. lixula)* and barbs (*S. granularis*). The spines of *P. lividus* are also shown to be the most compact with the wedges located very close to each other, whereas the wedges of *A. lixula* and *S. granularis* are more distant (Figure 3b, Figure 4b, Figure 5b). Observing the cross- section of the spines the main difference is that the porous zone seems to be relatively narrow in *P. lividus.* Since the sections of all spines were taken from the middle of the shaft, the porous zone may change in terms of surface width in different regions of the spine. Lauer et al. (2017) [36] concluded that the porosity of the spine increases from base to tip. The fact that the present study reported a very limited porous zone in the spine of *P. lividus* may be related to the fact that the cross section was taken from the middle of the shaft. It may occupy a wider area towards the tip. *A. lixula* the least number of wedges (14–15), followed by *P. lividus* (17), with *S. granularis* having the most and narrowest wedges (32) (Figure 3c, Figure 4c, Figure 5c). Note that the samples may still be filled with organic tissue which is most visible of the cross section of the spine of *A. lixula. A. lixula* was the only species that showed variety in the measurements of the number of wedges. *A. lixula* exhibited an outer imperforated cortex with an inner labyrinthic stereom regarding the spines. The cross-section of the spines of *S. granularis* showed two types of stereom, an outer galleried and an inner laminar stereom. Finally, the tests of the three sea urchins differ from one another, in that *P. lividus* and *S. granularis* present lines of secondary tubercles, which are absent in the test of *A. lixula*. In addition, when viewed in higher magnifications, different structures of stereom are visible (Figure 3d, Figure 4d, Figure 5d).

Such morphologic characteristics may play a role in the differentiation of the mechanical properties of the skeletons of the three species, since they change the structure and shape of both the test and spines the superficial surface of the plates of both *A. lixula* and *S. granularis* are characterized of perforate stereom. Wainwright et al. (1976) [37] showed that this type of stereom increases the resistance of the plate to bending stress. Another important function of this stereom is the increased protection against surface abrasion [15]. On the other hand, *P. lividus* exhibited a type of dense labyrinthic stereom. The labyrinthic stereom is the main architectural design used by most echinoids. The primary characteristic is the isotropic arrangement of the trabeculae, which ensures that the stereom is equally strong in all directions. Regarding the spines Grossmann and Nebelsick 2013 [38], report that the advantage of laminar stereom in the spines is that it has a low density and thus can be constructed relatively quickly compared to other types. They also concluded that the more chaotic distribution of struts in the labyrinthic stereom may serve to hinder the proliferation of cracks. Lastly in the spines of the three investigated species the septa seem to be the ones responsible for the mechanical stiffness while the porous zone towards the center of the spine is characterized of lower mechanical quality [39]. Mechanical tests with regard to the microstructural morphology should be carried out in order to better estimate the role of stereom differentiation in sea urchins. EDS analysis showed that spines and test are mainly composed of the same elements, but in different concentrations (Table 1; Figure 2). *P. lividus* exhibited significant differences between test and spines in all element concentrations except for S. *A. lixula* showed significant differences between the two investigated skeletal structures in Ca, Mg and Mg/Ca and *S. granularis* in Mg, Cl, Mg/Ca. Differences in the elemental concentrations between species occurred in Mg and Mg/Ca ratio both in the spines and tests while the only other significant differences were shown to be in Ca concentrations of the spines (Table 2). Possible temporal variations of the elemental concentrations were also observed, mostly in the test of *P. lividus* in Ca, Na, Cl and the Mg/Ca ratio. The spines of the same species showed to significant temporal differences. Regarding *A. lixula* significant differences were recorded in Na in the test and in S in the spines. Finally, the test of *S. granularis* exhibited different temporal concentrations in Mg and S, while the spines in Ca and S.

Changes in the chemical elements affected by time may be explained by various reasons, such as changes in abiotic factors (e.g., temperature, salinity, etc.), time-related diet changes or a combination of them. Hermans et al. (2011) [40] concluded that Mg/Ca ratio in the skeleton of sea urchins is defined by salinity levels, while also regulated by the presence of intrastereom organic matrix. Byrne et al. (2014) [33] showed that temperature also alters the Mg content in the skeleton of sea urchins. Kobuk et al. (2019) [41] showed that the Mg/Ca ratio is also regulated by food intake. *P. lividus*, being an opportunistic generalist, utilizes apostatic feeding, which allows it to switch from a preferred, but rare resource to a less preferred, but abundant one [42]. The same pattern may be true to a lesser extent for *S. granularis*, while *A. lixula* seems to have the most stable elemental composition throughout time. Finally, according to Hermans et al. (2011), the interstereom organic matrix seems to largely account for the fact that spines and tests have different elemental compositions.

As Figure 1 suggests, temperature in the selected sampling site reached highest values in summer, but also in September, while salinity apparently followed an antiproportional pattern, with lowest values recorded in summer. Since the elemental composition of the spines and tests of the investigated species does not follow a trend similar to temperature or salinity, it is indicated that other factors (e.g., vital effect, dietary preferences, etc.) also play key role. To explain the possible temporal differences in elemental composition observed in these species, more factors must be taken into account. Thus, further research should be conducted in order to shed more light in to how the chemical composition of the skeletons of these species changing according to time and also in to why *P. lividus* shows more variety in the elemental composition between test and spine, compared to the other two species (Table 1).

XRD analysis showed that the tests of *S. granularis* and *A. lixula* consist of the same form of magnesian calcite, whereas *P. lividus* seems to have twice the amount of Mg in the test. The spines of *P. lividus* are made up of a Mg-calcite with a lesser amount of magnesium compared to the spines of the other two species, more specifically about two times less (Table 3, Figure 7). Drozdov et al. (2016) [27] reported the presence of halite in the crystal of the skeleton of sea urchins in concentrations of 1% to 2%. The present study also reports the same mineral in concentrations of 3%–4%, however under no circumstances does this prove the biomineralization of halite by echinoderms. It is likely that this result occurred due to contamination (impurity from sea water, sample treatment with NaClO, etc.). Ma et al. (2009) [43] showed that Mg content in the grinding tooth increases towards the tip, and they hypothesized that higher Mg content enhances the mechanical hardness of the calcite. However, the hardness of the calcite is not the only factor affecting the durability of the skeleton under mechanical pressure. Microstructural features also play a significant role in the tenacity of the skeletal elements under tension. Mg content, however, also increases the solubility of the Mg-calcite [20,44,45,46]. This is the main reason organisms like sea urchins, which are now mainly relying on their mechanically durable, Mg-rich calcitic skeleton, may face the heaviest consequences of the ocean acidification effects. Elemental analysis showed that the tests have a higher Mg:Ca ratio than the spines, which may make the test more vulnerable to dissolution due acidification. Furthermore, EDS results showed that *S. granularis* has the highest Mg concentrations both in the test and the spine, while also having the highest Mg:Ca ratio in both skeletal structures. However, XRD results determined that the test of *P. lividus* has the highest content of Mg in the calcite. In both methods the spine of *P. lividus* was found to have the lowest percentage of Mg. It should be noted that the difference between the results of XRD and EDS analysis is to be expected, since the first method deals only with the concentrations of Mg and Ca in the compound biomineral (Mg-CaCO_3_), while the latter describes the concentration of the chemical elements in the investigated skeletal parts in general. However, live sea urchins utilize their physiology in order to adapt to different environmental conditions, which means that Mg content in the calcite alone is not a valid indicator to assess the effects of ocean acidification [18]. In any case ocean acidification effects will vary with species. The absence of urchins around some vent sites (pH 7.6–7.8), indicates that this level of acidification may prevent survival of sea urchins potentially due to vulnerability of a weaker skeleton to predation. However, this trend differs among sea urchin species as shown in a recent study where Arbacia lixula was particularly abundant at vent sites, while Paracentrotus lividus was not [47,48,49].

## 5. Conclusions

Morphologic differences in the skeletal elements of the three species hint to different mechanical properties. *P. lividus* seems to be more affected by time-related changes, compared to the other two species. Mg is the only element, which was never affected by time, in all three species. The lowest Mg content was founded in the spines of *P. lividus* ranging from 0.22% to 0.4%. The solubility of Mg-rich calcite increases with higher concentration of Mg, which could mean that the spine of *P. lividus* is the least vulnerable skeletal element, while the test may be the most susceptible to ocean acidification. These observations may play a decisive role for the balance of the ecosystems, where these species coexist. However, more research must be conducted to evaluate how ocean acidification will affect the Mediterranean sublittoral benthic ecosystems. It should be noted that the experiments in the present study were not conducted with regard to seasonal growth since the specimens were not labeled. Thus, the results are preliminary and do not determine the effect of seasonality. More research should be conducted in order to estimate the effects of season in the chemical composition of the skeleton of the investigated species.

## Figures and Tables

**Table 1:** One-way ANOVA and Kruskal–Wallis results for the elemental concentrations of spines and tests between *A. lixula*, *P. lividus* and *S. granularis*. Significant differences (*p* < 0.05*)* are highlighted in bold. (F—variance of the group means for ANOVA; H—variance of the group means for Kruskal–Wallis; *p*: probability value).

Chemical Elements		*p*	F or H
Ca	Test	0.5861	H = 1.069
	Spine	**0.001**	H = 13.767
Mg	Test	**0.0168**	H = 8.19
	Spine	**0.0001**	H = 39.674
S	Test	0.172	H = 3.511
	Spine	0.3389	F = 1.103
Na	Test	0.3323	H = 2.204
	Spine	0.5761	F = 0.5568
Cl	Test	0.0952	H = 4.705
	Spine	0.1356	H = 3.996
Mg/Ca	Test	**0.047**	H = 6.08
	Spine	**0.0001**	H = 40.538

**Table 2:** Results of one-way ANOVA and Kruskal–Wallis test regarding the temporal variations of each element in the spines and test of *A. lixula*, *P. lividus* and *S. granularis*. Significant differences (*p* < 0.05) are highlighted in bold. (F—variance of the group means for ANOVA; H—variance of the group means for Kruskal–Wallis; *p*: probability value).

Chemical Elements		*A. lixula*		*P. lividus*		*S. granularis*	
		*p*	F or H	*p*	F or H	*p*	F or H
Ca	Test	0.7543	F = 0.4009	**0.001**	F = 9.036	0.479	F = 3.280
	Spine	0.07	F = 2.84	0.19	F = 1.738	**0.038**	F = 3.553
Mg	Test	0.2766	F = 1.409	0.207	F = 1.7	**0.0053**	F = 6.206
	Spine	0.479	F = 0.865	0.11	F = 2.269	0.1286	F = 2.193
S	Test	0.379	H = 3.081	0.145	H = 5.389	**0.044**	H = 8.056
	Spine	**0.013**	F = 4.847	0.13	F = 2.15	**0.01**	F = 11.311
Na	Test	**0.03**	F = 3.546	**0.0003**	F = 11.368	0.618	F = 2.996
	Spine	0.772	H = 1.119	0.4678	H = 2.542	0.6366	F = 0.58
Cl	Test	0.103	F = 2.424	**0.0001**	F = 16.404	0.4529	H = 2.621
	Spine	0.2518	F = 1.503	0.3357	H = 3.387	0.93	H = 0.4222
Mg/Ca	Test	0.7294	F = 0.4373	**0.0002**	F = 12.576	0.1653	F = 1.931
	Spine	0.5873	F = 0.6622	0.419	F = 0.9983	0.0587	F = 3.055

**Table 3:** Data of an X-ray powder diffraction of tests and spines of the three species.

Sample		Chemical Composition of Crystalline Phase
*A. lixula*	Test	Magnesian calcite (Mg_0.06_Ca_0.94_)CO_3_, NaCl
	spine	Magnesian calcite (Mg_0.064_Ca_0.936_)CO_3_, NaCl
*P. lividus*	Test	Magnesian calcite (Mg_0.129_Ca_0.871_)CO_3_, NaCl
	spine	Magnesian calcite (Mg_0.03_Ca_0.97_)CO_3_, NaCl
*S. granularis*	Test	Magnesian calcite (Mg_0.06_Ca_0.94_)CO_3_, NaCl
	spine	Magnesian calcite (Mg_0.06_Ca_0.94_)CO_3_, NaCl
